# Medicaid Waivers and Tenancy Supports for Individuals Experiencing Homelessness: Implementation Challenges in Four States

**DOI:** 10.1111/1468-0009.12514

**Published:** 2021-04-27

**Authors:** FRANK J. THOMPSON, JENNIFER FARNHAM, EMMY TIDERINGTON, MICHAEL K. GUSMANO, JOEL C. CANTOR

**Affiliations:** ^1^ Rutgers Center for State Health Policy; ^2^ Rutgers School of Public Affairs & Administration; ^3^ Rutgers School of Social Work; ^4^ Rutgers School of Public Health

**Keywords:** Medicaid, homelessness, Section 1115 demonstration waivers, policy implementation

## Abstract

**Policy Points**

- Medicaid policymakers have a growing interest in addressing homelessness as a social determinant of health and driver of the potentially avoidable use of expensive medical services.
- Drawing on extensive document reviews and in‐depth interviews in four early‐adopter states, we examined the implementation of Medicaid's Section 1115 demonstration waivers to test strategies to finance tenancy support services for persons experiencing or at risk of homelessness.

**Context:**

The Affordable Care Act extended Medicaid eligibility to large numbers of individuals experiencing or at risk of homelessness. This legislative development and the growing recognition of homelessness as a significant social determinant of health have encouraged advocates and policymakers to seek new ways to use Medicaid to provide housing supports.

**Methods:**

We conducted 28 semistructured interviews with 36 stakeholders in four states. The stakeholders were government administrators, health care providers, nonprofit housing staff, and consultants. We supplemented these interviews with extensive reviews of public documents, media accounts, think‐tank reports, and published literature. We also conducted a systematic inductive qualitative analysis.

**Findings:**

We identified seven challenges to the successful implementation of tenancy support demonstration projects: resolving the housing supply and NIMBY, removing silos between health care and homeless services providers, enrolling and retaining the target populations in Medicaid, contracting with and paying tenancy support providers, recruiting and retaining key workers, ensuring Medicaid's waiver durability, and reducing administrative crowd‐out and waiver burden.

**Conclusions:**

Notwithstanding these challenges, three of the four states have made significant progress in launching their initiatives. At this point, the fourth state has delayed its start‐up to consider alternatives to a Medicaid demonstration waiver to provide tenancy supports. The experience of the four states suggests lessons for Medicaid officials in other jurisdictions that are interested in pursuing tenancy support initiatives. Nevertheless, the limitations of tenancy support waiver programs suggest that federal policymakers should consider allowing states to more directly subsidize housing costs for those experiencing or at risk of homelessness as an optional Medicaid benefit.

An estimated 568,000 people in the united states experience homelessness on a given day, with a little more than 60% of them finding some respite in emergency shelters or transitional housing. The remaining 211,000 individuals are “unsheltered.”^1^ These statistics do not include those at significant risk of homelessness because of eviction or release from an institution, such as a hospital or prison. Homelessness and poor health march in lockstep. People with health problems such as mental illness or substance use disorder (SUD) are more apt to become homeless. In turn, being without stable housing increases the probability of morbidity and mortality. For instance, a report from the National Academies of Sciences, Engineering, and Medicine found that individuals experiencing homelessness are at greater risk for infectious diseases, serious traumatic injuries, drug overdoses, violence, and death due to extreme heat or cold.^2^ ^(p25)^ The COVID‐19 pandemic is likely to increase housing instability and homelessness, imposing grave new health risks on those without adequate shelter.

Homelessness not only undermines health but also drives up health care costs, primarily through higher rates of emergency department and hospital utilization.^2^ For example, a recent study focused on New Jersey found that the health care costs of Medicaid enrollees experiencing chronic homelessness were as much as 27% greater than those of a comparison group of demographically and clinically matched beneficiaries who had not experienced homelessness. The study also found that individuals with behavioral health or physical disabilities who had experienced only a modest amount of time without a suitable place to live had greater emergency room utilization and ambulatory care sensitive admission rates than the comparison group.^3^

These and similar findings have kindled widespread interest in supportive housing initiatives for people experiencing homelessness as a pathway to more cost‐effective health care. These initiatives assume various forms, from those helping people locate stable housing and apply for rental subsidies to far more comprehensive approaches combining housing, health care, and social services. In regard to the latter, permanent supportive housing efforts typically integrate rental assistance with a variety of tenancy supports, health care, and social services, which can be of indefinite duration. Tenancy supports incorporate pre‐tenancy activities such as the search for appropriate housing and assistance with documentation requirements. These supports also include tenancy‐sustaining activities such as landlord dispute resolution and anger management. One supportive housing model of growing interest to policymakers is “housing first.” According to the US Department of Housing and Urban Development (HUD), which has endorsed this approach for its grant programs, housing first emphasizes that “persons experiencing homelessness should not be screened or discouraged from [housing] programs because they have poor credit history, or lack income or employment. Additionally, people with addictions to alcohol or substances should not be required to cease active use before accessing housing and services.”^4^

A key question is, of course, whether and the degree to which supportive housing initiatives, when accompanied by intensive case management and coordinated care, have positive effects. In this regard, the National Academies has cautioned that major research gaps exist and the contribution of housing support initiatives to improved health and reduced health care costs has not been shown conclusively. Nor does the available evidence document that housing initiatives are budget neutral; that is, that reductions in health care costs pay for the additional spending needed to launch and sustain the initiatives.^2^ Still, recent studies of supportive housing have demonstrated the potential for less use of emergency departments and hospitals as well as less spending on this cohort.^3^, ^5^, ^6^, ^7^Research has also shown a connection between supportive housing and access to care, self‐reported mental health outcomes, and overall well‐being.^8^, ^9^, ^10^Moreover, the National Academies, while calling for additional research, has affirmed that “housing in general improves health.”^2^ ^(p4)^

Encouraged by such findings, Medicaid officials in several states have moved to provide various supports to those facing housing insecurities. In doing so, they face formidable barriers embedded in federal Medicaid regulations and guidance.^11^, ^12^ The program has long subsidized certain housing for seniors and younger people with disabilities, primarily in skilled nursing homes, intermediate care facilities, and small group homes. But it prohibits payment for housing for other cohorts, including the chronically homeless. Nonetheless, as of early 2020, 11 states had received approval from the Centers for Medicare and Medicaid Services (CMS) for tenancy support initiatives that at least in part targeted homelessness. Six additional states had, in varying degrees, moved toward obtaining CMS sign‐offs on such ventures.^13^ In pursuing these initiatives, officials face myriad challenges of program design and implementation. Research has repeatedly shown that implementation processes markedly shape the fortunes of public initiatives.^14^ To advance our understanding of the implementation challenges associated with Medicaid housing initiatives, we examined the experience of four states (California, Illinois, Maryland, and Washington) that at an early point (2016 to 2018) obtained Section 1115 demonstration waivers to pursue tenancy supports.

We next describe the Medicaid policy context of these waivers and their key design features. Following a discussion of methodology, we assess seven core implementation challenges and the degree to which the four states surmounted them. In addition to acknowledging our research limitations, our concluding section addresses two principal questions. First, what did we learn from our research that may help other states effectively launch and implement Medicaid housing initiatives? Second, what changes in Medicaid policy should we consider in order to better factor in housing as a social determinant of health?

## Context and Design in Four Waiver States

While policy barriers to Medicaid housing supports persist, the Affordable Care Act of 2010 (ACA) enhanced opportunities to address homelessness. Before the ACA, the Supplemental Security Income (SSI) program for low‐income people with disabilities was the main route for persons experiencing homelessness to obtain Medicaid. (Mental illness, but not substance use disorder [SUD], often enabled a person to qualify.) The ACA Medicaid expansion gave eligibility to adults aged 19 to 64 and with an income below 138% of the poverty line. This is a cohort with significant numbers of individuals experiencing or at risk of homelessness. By early 2020, 36 states and the District of Columbia had expanded Medicaid. More modestly, the ACA also increased opportunities to fight homelessness by amending Section 1915(i) of the Social Security Act to give states somewhat greater flexibility to provide tenancy supports to certain individuals with mental illness and SUD through a state plan amendment rather than a waiver.

In this new policy context, several states saw Medicaid Section 1115 waivers as a promising tool for extending tenancy supports. Authorized in 1962, the use of these demonstration waivers had mushroomed in the period starting with the Clinton administration. Roughly 80% of the states have these waivers, with about one‐third of federal Medicaid expenditures supporting waiver‐based activities.^15^ A 2015 CMS bulletin drew attention to this well‐known tool, among others, for providing Medicaid tenancy supports. The bulletin described the housing services that Medicaid could subsidize to assist “individuals already in the community” rather than those leaving a long‐term care facility (the program's perennial focus).^11^ The bulletin listed a spectrum of pre‐tenancy and tenancy‐sustaining services that the federal Medicaid program would fund. The Trump administration, despite its generally trying to erode enrollment of the Medicaid expansion cohort,^16^ nonetheless signaled support. Both Secretary of Health and Human Services Alex Azar^17^ and CMS Administrator Seema Verma^18^ endorsed greater Medicaid flexibility to address housing needs as a social determinant of health. The four study states were among the first responding to CMS's signals of receptivity to tenancy supports for those experiencing homelessness. California received authorization to launch its five‐year demonstration in 2016, Maryland and Washington in 2017, and Illinois in 2018.

The four waiver states varied appreciably in the magnitude of their homelessness problems, as Table 1 shows. California and Washington have more acute challenges with homeless populations per ten thousand people that are well above the national average of 17. In contrast, Illinois and Maryland had per capita rates well below the national mean. The seriousness of the homelessness problem in California is striking. More than a quarter of the nation's homeless population lives in this state, and its per capita homeless rate is more than double the national average. California also stands out in the proportion of its homeless cohort who are “unsheltered.” More than 70% of that state's homeless individuals fall into this category, compared with 19% in Illinois, 21% in Maryland, and 44% in Washington.

**Table 1:** Homelessness in Four States With Medicaid Tenancy Support Waivers, 2019

Jurisdiction	Total Homeless Individuals	% of Total	Homeless Individuals per 10,000 Population	% of Homeless Individuals Who Are Unsheltered
California	151,278	27%	38	70%
Illinois	10,199	2%	8	19%
Maryland	6,561	1%	11	21%
Washington	21,577	4%	29	44%
United States	567,715	100%	17	37%

Data from HUD Exchange 2019 continuum‐of‐care homeless population and subpopulation reports.

Even though the four waiver initiatives differ in myriad ways, they do share some features. With variations, all the waivers target Medicaid enrollees with some combination of the following characteristics: (1) they are experiencing or at risk of becoming homeless (e.g., while transitioning out of hospitals, behavioral health facilities, jails, or nursing homes); (2) they are precariously housed in the broader community and at risk of institutional placement; (3) they have serious health problems such as chronic physical conditions or behavioral health issues; and (4) they have repeated instances of the avoidable use of emergency departments and inpatient hospitals. All the waivers call for the provision of pre‐tenancy and tenancy‐sustaining services to the target populations.

Although the waiver initiatives shared these features, they varied considerably in their formal structural arrangements to provide homeless services. As Table 2 indicates, California and Maryland pursued a *locally driven intergovernmental model*, which depended on localities to develop effective approaches to providing housing supports. In both states, the requests for proposals (RFPs) required, among other things, local applicants to document their need for Medicaid tenancy supports and to demonstrate that they had commitments from diverse stakeholders (e.g., in the health care, housing, justice communities) to address the problem. The localities had to commit to credible performance measurement and reporting systems. They also had to demonstrate their ability to provide local funds to the state to generate the federal Medicaid match needed to subsidize their tenancy support activities. (In essence, the locality would transfer funds to the state to spend on Medicaid tenancy supports. In turn, the federal government would match the money the state spent. Assuming the locality lived up to its commitment to provide tenancy supports for Medicaid enrollees, it would recapture the money it originally contributed and receive the federal matching funds.)

**Table 2:** Selected Characteristics of Medicaid Tenancy Support Waivers^a^

Name	Structure	Participant Eligibility Criteria	Projected Numbers Served	Period of Waiver Approval
California: Whole Person Care Pilot	Locally driven intergovernmental; localities can apply to address the housing and other social needs of targeted Medi‐Cal enrollees; 24 pilots sponsored by county governments plus one from the city of Sacramento obtained state approval.	Individuals at risk of or experiencing homelessness who have a demonstrated medical need for housing or supportive services (including but not limited to repeated hospital visits, nursing facility placement, 2 or more chronic conditions, behavioral health disorders).	Not explicitly stated (cumulative total enrollment at the end of 2019 was about 160,000).	2016 to 2020
Illinois: Assistance in Community Integration Services	Ambiguous because the initiative did not take off; the waiver request indicated that the state planned to contract with community‐based nonprofits in the Chicago area to provide tenancy supports; in partnership with Medicaid MCOs, the housing providers would facilitate intensive case management and health care.	At least one health criterion (4 or more hospital admissions or ED visits per year; 2 or more chronic conditions) and at least one housing criterion (homeless or at imminent risk of institutional placement).	Annual Medicaid enrollees served as of 2023: 3,750.	2018 (July) to 2023
Maryland: Assistance in Community Integration Services Pilot	Locally driven intergovernmental; state Medicaid agency gave localities the opportunity to apply for funding to support tenancy support pilots; four localities won state approval to launch pilots: Baltimore City, Cecil County, Montgomery County, and Prince George's County.	At least one health criterion (4 or more hospital admissions or ED visits per year; 2 or more chronic conditions) and at least one housing factor (homelessness or at imminent risk of institutional placement).	300 Medicaid enrollees annually; subsequently amended to 600.	2017 to 2021
Washington: Foundational Community Supports	Third‐party administrator; state officials contracted with Amerigroup, a Medicaid MCO, to manage the initiative; it recruited nonprofits throughout the state to provide tenancy supports; it also attempted to secure the commitment of four other Medicaid MCOs to the housing initiative.	Health need (behavioral where support needed to prevent functional deterioration; health care (PRISM) score 1.5 or above indicating higher than average expected Medicaid expenditures); housing need (homelessness, history of institutional or adult residential placement; frequent turnover of in‐home caregivers).	Estimated annual monthly caseload of 7,500 for either housing or employment supports (data suggest about half receive tenancy assistance).	2017 to 2021

Abbreviations: ED, emergency department; MCO, managed care organization.

^a^ Material obtained from waiver documents.

The efficacy of the intergovernmental model substantially depends on the degree to which local governments submit proposals acceptable to state officials. Both California and Maryland had positive experiences. In California, 23 counties, one small‐county collaborative (consisting of three jurisdictions), and the city of Sacramento obtained state approval for Whole Person Care pilots. While the pilots were permitted to focus on social determinants other than housing, all of them, albeit in varying degrees, offered tenancy supports.^19^ ^(p26)^ Participating localities contained 85% of California's population and slightly greater rates of homelessness than did jurisdictions that did not join the pilot.^20^ For instance, Los Angeles County, with more than a quarter of the state's population and nearly 40% of its homeless individuals, established a pilot. In the Bay Area, major population centers like Alameda, San Francisco, and San Mateo counties participated. Nor were participants limited to urban areas. Shasta County, near the Oregon border, with 180,000 residents and a homeless population of roughly 700, also launched a pilot. California's waiver proposal did not explicitly predict the overall number of Medicaid enrollees who would receive tenancy supports. (By the end of 2019, the cumulative total enrollment in Whole Person Care was 160,000.)^21^

Maryland officials also elicited significant local government participation in their Assistance in Community Integration Services (ACIS) initiative. Three urban jurisdictions—Baltimore City along with Montgomery and Prince George's counties—participated, as did rural Cecil County in the northern part of the state. While fewer than 20% of Maryland's 24 counties joined, the four participating jurisdictions were home to nearly half the state's population. These localities also had the lion's share of Maryland's homeless population. The city of Baltimore alone claimed nearly 40% of this cohort. Combined, the participating jurisdictions encompassed close to 60% of the state's homeless population. The waiver proposal initially estimated that ACIS would serve 300 Medicaid enrollees annually.

Washington adopted a *third‐party administrator* approach to its housing initiative. The state issued an RFP^22^ targeting a business or nonprofit organization to implement the state's Foundational Community Supports initiative, which seeks to provide tenancy or employment services to specified Medicaid enrollees. The RFP envisioned that the successful bidder would serve an average monthly caseload of 7,500 individuals and would contract with housing and other pertinent providers to deliver services. State officials also expected the third‐party administrator to obtain the cooperation of the state's five Medicaid managed care organizations (MCOs) to serve the target population and establish a “sustainable model” for tenancy supports after the waiver ended. Amerigroup, one of the state's five Medicaid MCOs, won the bid, pledging to run the initiative out of an administrative division distinct from those serving its own Medicaid enrollees and to secure the participation of the other MCOs. By December 2019, Amerigroup had contracted with 100 entities to provide tenancy supports at 301 sites throughout the state.^23^

The design embedded in the Illinois Assistance in Community Integration Services initiative remains unclear, since it had not launched as of 2020. The waiver documents, however, suggest the possibility of a direct *community‐based contracting model*. As initially envisioned, this model called for the state Medicaid agency to contract with community‐based nonprofits in the Chicago area to provide tenancy services to enrollees who were experiencing or at risk of homelessness. The nonprofits would coordinate their efforts with several Medicaid MCOs. The state projected that the number of enrollees served annually would grow to 3,750 in 2023. For reasons we will discuss later, Medicaid officials never issued guidance that more precisely defined the qualifications that nonprofit homeless services providers had to possess. It is also possible that Illinois, like Washington, would have opted for a third‐party administrator model if it had moved forward. A work group set up to advise state officials recommended this approach.^24^

## Methodology

Our sample of four states represents the preponderance, if not the entirety, of early‐adopter jurisdictions with Section 1115 waivers explicitly focused on tenancy supports for people experiencing homelessness and housing insecurity. All four had obtained approval for these demonstration waivers by mid‐2018, providing fertile ground for analyzing start‐up implementation challenges. Several other states have since decided to pursue tenancy supports through Medicaid demonstration waivers or state plan amendments. But these initiatives are still in the early stages.

Our study rests on a qualitative, inductive methodology. We used rapid assessment procedures, an approach focused on categorizing and characterizing targeted domains, which is particularly helpful for describing operations and identifying areas for improvement in health‐related programs.^25^ In order to provide pertinent contextual background on the demonstrations and help identify interview subjects, we employed expert consultants who were knowledgeable about homeless services and Medicaid issues in each of the study states. These consultants reviewed our findings and provided feedback to help correct any errors of fact or interpretation. In 2019, we conducted 28 semistructured interviews with 36 key stakeholders in the four states who were familiar with the demonstrations. These stakeholders included government administrators, health care providers, consultants, and staff providing tenancy supports (some of whom worked for nonprofits that also developed or maintained housing). The interviews, averaging about one hour in length, were recorded and transcribed. They were independently coded by two members of the research team, who reached agreement on the key themes using a consensus coding process.^26^ Our analysis also drew on an extensive review of public documents, media accounts, think‐tank reports, and the research literature on health care, homelessness, and housing.

## Key Implementation Challenges

The stakeholders we interviewed described a bevy of implementation challenges that needed to be addressed to achieve the objectives of the tenancy support waivers. In coding the interviews, the seven challenges described next generally met one major criterion: at least half (and often more) of the 28 interviews cited them. The sole exception is the seventh challenge, which draws exclusively on Illinois stakeholders to account for the state's stalled takeoff.

### Challenge 1: Housing Supply and NIMBY

There's no freaking housing… . We are having a historically ridiculous housing price rise. (Interview 11, public official)

Although we like to think of ourselves as rather politically progressive, we have a lot of siting problems. Even if we were going to, for example, try to do a congregate living environment where we could somehow provide supports on site … we could never get that past our city council. (Interview 23, public official )

Nearly all those interviewed identified a lack of suitable, affordable housing as a major implementation challenge. Their comments suggested that this supply issue had at least four dimensions. The first, captured by the first quotation, had to do with spillovers from a general shortage of affordable housing for low‐income renters in all four states (especially California and Washington).^27^ This basic shortage in turn compounded the task of finding accommodations for clients experiencing homelessness.

A second, more immediate supply problem pertained to the limited financial support to subsidize housing for targeted enrollees. Since the waivers did not permit Medicaid to pay the rent or otherwise provide housing, the demonstrations needed to obtain assistance from local public housing authorities or other sources. Most housing authorities depend heavily on HUD's Section 8 vouchers to assist those experiencing or at risk of homelessness.^28^ Sometimes the housing authorities were a valuable source of support. As one official noted: “It was essentially us going to [the housing authority] and saying ‘Hey, we got this big new pilot. We need 40 something subsidies,’ and they would just give them to us” (Interview 8). More often, the housing authority could not supply all the needed vouchers, with the demand for them greatly outpacing their availability and some applicants remaining on waiting lists for years. To compound these problems, the stakeholders noted the reluctance of some landlords to accept Section 8 vouchers, especially in a tight housing market (a problem that has been found in other studies).^29^, ^30^ One homeless services provider told us that to overcome landlords’ unwillingness to rent to those with HUD vouchers, state policymakers had established a fund that would reimburse them for damage to their properties caused by subsidized renters.

The limits to support from public housing authorities prompted the demonstrations to seek other funding sources. Stakeholders in California noted that the state and several counties had approved tax measures to create flexible housing subsidy pools to assist those experiencing homelessness, and more than half the California pilots drew on these pools.^19^ ^(p245)^ Several respondents also mentioned their efforts to help enrollees qualify for disability payments, which could then be applied to rents. Others expressed the hope that MCOs, hospitals, and developers might become a funding source for housing. Despite these efforts, the respondents reported many instances in which they provided tenancy supports to those experiencing homelessness without finding them suitable accommodations.

Third, some respondents referred to supply problems created by the special needs of many of the homeless individuals and the historical reluctance of local officials to approve housing tailored to these needs. Housing of otherwise reasonable quality still might not be suitable for clients with particular health problems (e.g., an inability to climb stairs) or who need to be near public transportation. So, too, as suggested by the second interview quoted at the beginning of this section, local officials have often been averse to approving special housing facilities for people experiencing homelessness.

Finally, stigma and NIMBY contributed to the scarcity of housing. In part, this was manifested in a legacy of resistance to the physical siting of facilities designed to aid people experiencing homelessness, such as congregate living structures, shelters, and sobering centers. Other issues involved neighborhood acceptance of clients into existing housing. One tenancy support provider from a more affluent county observed “that the folks we're housing may not look like [their neighbors], act like them. They might have different interests. So, we do have one person in particular who likes to dress in military garb and, boy, did he alarm his neighbors.” The provider indicated that one of its major tasks was to change “those stigmas and those stereotypes” (Interview 24). At times, neighbors file complaints with landlords or the police for behavior they find odd or suspect because it violates their sense of normal behavior, not because it breaks tenancy rules or local ordinances.

A handful of respondents pointed to race as a factor contributing to housing problems. One public official suggested the presence of “structural racism… . The percentage of the people that we're working with that are homeless who are Black is just really striking and horrifying… . It makes it easier for people … [to feel] like it's somebody else's problem” (Interview 11). In a similar vein, another official observed:

A number of our clients are African American, and they're overrepresented. Then when we try to put them in housing units that don't look like them, we've seen barriers and … lots of complaints over very silly things. Like the person was smoking outside… . We do experience a lot of prejudice with our folks. (Interview 23)

### Challenge 2: Breaking Down Silos Between Health Care and Homeless Services Providers

We all know the health care system is very fragmented and confusing, but the housing world is far worse. It could be a full‐time job for me just learning the rules, regulations, and who gets a voucher and who doesn't. (Interview 7, health care provider)

It took a really long time for me to really settle into the language of health care and know what people were talking about … probably a year for me to feel that I could even have a conversation about this. And I think the same is on the other side. The folks that we're talking to for the first time on the health care side really don't know anything about housing. (Interview 21, homeless services provider)

Housing services folks … think that health care has a lot of money, and so they tend to be resentful of efforts to pull their work into health settings. Just getting over the cultural divide that comes between these scrappy, multiply tattooed and pierced housing warriors to bring them into work amicably with the social workers in the hospital who are trying to discharge people. That cultural divide has been a big deal. (Interview 11, public official)

As these quotations suggest, breaking down barriers to communication and coordination between health care and providers engaged in homeless services is far from simple. Historically, the two groups of providers have inhabited different and relatively insulated silos. Each silo featured different clusters of actors with distinct educational and professional backgrounds. One contained HUD, local housing authorities, nonprofit tenancy support providers, and developers, among others. The other was composed of CMS, state Medicaid agencies, MCOs, hospitals, and other health care providers. Each silo encompassed a distinctive set of federal, state, and local policies; administrative regulations; and standard operating procedures. Each had a distinctive information system that made the practical exchange of data for operational purposes hard to achieve. Each incorporated different approaches to financing and paying for services. And each had its own set of values, norms, and beliefs about how to get things done. (Though not the focus of this article, housing developers exist in yet a third silo, in which they think about financing, tax credits, and how to make affordable housing a reasonable investment.)

The prospect of receiving Medicaid funding to subsidize tenancy services strongly incentivized housing nonprofits to participate in the demonstrations. But this did not necessarily eradicate their concerns about such involvement. For instance, one employee of a homeless services nonprofit worried that “dabbling into Medicaid will sort of be a slippery slope … down the path of being a medical provider” (Interview 21). Many in homeless services did not look forward to the time and effort involved in learning “Medicaid speak” (Interview 3). To be sure, these reservations did not apply to some of the larger housing nonprofits that had previously worked with Medicaid. One such organization had begun years ago as a facilitator of housing for Medicaid enrollees with intellectual and developmental disabilities. Another large housing provider also delivered mental health services to Medicaid beneficiaries. But the demonstrations also sought to involve smaller community‐based homeless services providers that lacked Medicaid experience.

In turn, state Medicaid agencies and the MCOs with which they contracted often had a limited understanding of the nexus between housing and health care. The fact that in the past the waiver states had carved out behavioral health services from the domains of their primary Medicaid agencies and assigned them to other state bureaucracies magnified this problem. So too did the tendency to remove behavioral health care from Medicaid MCO contracts. Some stakeholders believed that these carve‐outs impeded efforts to integrate services to chronically homeless Medicaid enrollees, who disproportionately suffered from mental health and SUD issues. As one stakeholder put it, the concept of housing support initiatives is “very foreign” to the staffs of Medicaid agencies. They

have struggled to understand what it means to have a system of care that incorporates social services support as well as behavioral health care… . That is a very big hurdle to overcome, because we have always had a system of care … where behavioral health has been carved out. (Interview 1)

Among our four study states, Washington had attempted to address this problem through a major administrative reorganization. But at least initially, it had not resolved all the tensions between stage agencies representing physical and behavioral health perspectives, respectively. One health care provider involved in the housing support demonstration noted that state agencies were “giving us different messages. It makes for a convoluted message to us, and we're just trying to appease the different state agencies” (Interview 13).

In acknowledging the silo problem, several respondents also suggested ways to surmount it. One stakeholder observed, “Those silos can be bridged … through … administrative meeting structures and oversight structures, … specific policy and procedures that link together how they do things … and then data systems that can get them all information from each other” (Interview 6). At times, state Medicaid officials went out of their way to create such integrating structures. California officials, for instance, required localities to create diverse networks of participants for the Whole Person Care initiative. Local applicants had to designate a lead entity, at least one Medicaid MCO, a minimum of two community‐based partners, and public agencies responsible for delivering health services, behavioral health treatment, and housing supports. The approved pilots readily met and often exceeded this standard, with 20 to 30 network participants. For example, one pilot had 21 participants, including nine different county agencies, three municipal human services departments, three Medicaid MCOs, and six community‐based housing or health care organizations.

Some respondents pointed to concrete examples of constructive working relationships between health care and homeless services providers. One public official on a steering committee to assist homeless enrollees described the initial tensions between these two cohorts of providers: Health care providers would insist: “We've got to house these people that are on the streets and they're going to die if they don't get housed right now.” In turn, the housing provider knows that “we have only this much housing stock and none of it matches this client's needs.” The official acknowledged that

our housing provider understood better than we did why certain clients couldn't be housed in certain locations, having a lot to do with their medical conditions. They needed accommodations in the shower. They needed first floor access… . Those of us … on the clinical end didn't at first understand the complexity of the physical needs of many of those experiencing homelessness. (Interview 23)

### Challenge 3: Enrolling and Retaining the Target Population

It's very difficult for [the homeless] to get on and stay on Medicaid because a lot of people … are unsheltered, which means that you really need staff to do outreach, to go out into the streets and really find people and get them enrolled. (Interview 1, consultant)

The Medicaid program has long faced take‐up issues resulting in a significant proportion of those who could qualify for the program not being enrolled.^31^ ^(pp70‐100)^ Enrollment challenges for the tenancy support waivers extended beyond those ordinarily faced by Medicaid officials. This partly stems from difficulties in locating clients and documenting their eligibility. It also emanates from the need for enrollees to meet additional eligibility criteria. Those receiving tenancy supports not only need to enroll in Medicaid, but also must meet certain health care criteria (e.g., have certain chronic conditions and be frequent users of hospital emergency rooms or inpatient services). They also must meet certain standards for being homeless or precariously housed, as heavily influenced by HUD. In practice, this meant that those implementing the demonstrations needed access to both Medicaid and housing information systems.

The problems of locating clients stem from the fact that many are not in shelters and lack fixed addresses. In order to cope with this problem, project administrators rely on assistance from multiple entities. Referrals from hospital discharge and emergency departments play a role as do criminal justice agencies. One local official described a partnership with a special unit of the sheriff's department: “What used to happen is that we would hear … they were homeless, and by the time we tried to reach them they were gone… . Now the sheriff team will actually have the [individual] with them and give us a call” (Interview 7). In a similar vein, another administrator observed: “So our police impact teams [are] familiar with the encampments within the city, and they know that this particular individual is here on this day, or … saw them a week ago, and we can check in on them. So that creates a warm handoff into the program” (Interview 18). Government officials acknowledged the value of community‐based nonprofits in tracking enrollees. One local government had initially relied on its own employees to locate homeless clients. But administrators were reluctant to send this staff “into the homeless encampments by themselves because that can be unsafe.” Officials then contracted with a nonprofit that had a record of providing services in these encampments. This enabled them to move from a 3% rate of locating clients when they started to a 50% rate subsequently (Interview 18).

Many of those identified as meeting the eligibility criteria for tenancy supports cannot be promptly placed in housing or in shelters. Consequently, the problems of keeping track of clients persist, and numerous respondents talked about the “churning” of enrollment as a significant problem. Some clients die. Others leave the jurisdiction or lose touch with their caseworkers for other reasons. Still other stakeholders pointed to the administrative burdens of Medicaid redetermination processes as triggering disenrollment. Providers in Washington State expressed concerns that these redeterminations had to be carried out every six months rather than annually.

Documentation challenges compound those fueled by the need to locate and track clients. As one local official observed, “Sometimes individuals who are experiencing homelessness don't have any place to store documents, or store letters and things like that, and they are constantly having to redo … and get everything multiple times, multiple iterations” (Interview 18).

The need to meet HUD's eligibility criteria for homeless services was another significant take‐up challenge. As a major funder of local housing programs, HUD heavily influences both the eligibility criteria for homeless services and the system used to track those receiving them. Under the banner of providing a “continuum of care,” HUD has stressed the need for localities to offer a “coordinated entry process” to identify those who should receive housing subsidies and has even published a guidebook to facilitate these efforts.^32^ One primary purpose of coordinated entry is to ensure that those persons with the most acute care and housing needs—the chronically homeless—receive priority for permanent supportive housing. The agency defines chronic homelessness as being without housing for at least 12 continuous months or having four or more episodes of homelessness totaling at least 12 months over three years. Chronic homelessness also requires that a provider certify the presence of a disability that interferes with the capacity to be stably housed. These HUD criteria add to the burdens of documenting clients’ eligibility for tenancy supports. One local official noted that documenting the “chronicity of homelessness [is] … a lot of work.” An outreach worker might “need to go to that police officer who saw them homeless in front of the 7‐Eleven on this particular day, or go to the Social Security office and speak with that … person who can verify that they were homeless when they were trying to get this payment” (Interview 18).

To target clients for tenancy supports, local continuum‐of‐care entities, following HUD guidelines, establish homeless management information systems (HMIS), which incorporate client‐specific data on those using homeless services or at risk of or experiencing homelessness. The stakeholders identified challenges in implementing these HUD systems. As one local Medicaid official noted, we have “invested a lot in accelerating the standing up of our local coordinated entry systems, a lot into the homeless management information system rollout here. And we have invested in a lot of training.” Despite these efforts, the official commented that the rollout of the HMIS system has been “slow.” While making progress in “putting in place the infrastructure … we're not going to be where we want to be at the end” of the waiver (Interview 11).

While providing guidelines for HMIS systems, HUD does not mandate that their data be part of a statewide system. Some stakeholders viewed this lack of uniformity as problematic, with one state official noting that this fragmentation “just frankly drives me nuts.” Reporting has “really varied from local entity to local entity” (Interview 3). Another official saw HMIS fragmentation as a take‐up problem because the homeless are often transient.

So if you have someone … and they were homeless in [X] county and they came to [my] county, we would not know they were homeless in [X] county unless we actually picked up the phone to speak to our counterpart [there]… . We need to have a system, at least for the state … to see the larger picture. (Interview 15)

Other stakeholders expressed concerns about the degree to which HUD systems target clients that the Medicaid demonstrations can serve. One consultant observed:

I think [that] … coordinated entry functions that [HUD] has been creating to try to identify homeless people in a unified, organized way has been challenging so that many of the people on the coordinated entry lists are not necessarily the people who are particularly sick, which would make it hard to align [Medicaid] funding with the coordinated entry. I think that's been a major problem. (Interview 2)

Those identified and entered into the HMIS system may not meet the emergency room and hospital use criteria needed to enroll in the demonstration. The absence of an integrated system that simultaneously provides Medicaid and housing information about potential clients can yield take‐up inefficiencies. One homeless services provider, for example, complained about not having ready access to Medicaid utilization scores for the homeless individuals they were attempting to enroll, leading to a substantial rejection rate among those they proposed to serve. “So that scares me to have to go through all that paperwork … and just find out that they are not going to qualify. That just doesn't seem like a good system” (Interview 21).

A few stakeholders also questioned whether HUD criteria excessively restricted access to services under the waivers. One provider observed: “The HUD chronic homelessness criteria are pretty strict… . You can really easily not meet these HUD criteria, even though you really are very vulnerable and have experienced very long periods of homelessness” (Interview 19). A health care provider also said that targeting the most acutely disadvantaged tended to crowd out a prevention model focused more on those who were on the “precipice of becoming homeless” because of eviction or other problems. Attention to this cohort can lead to “tremendous cost savings from an initial small investment” (Interview 27).

### Challenge 4: Contracting With and Paying Homeless Services Providers

I think really on the fiscal side, just some of the contracting delays and getting to a place where all fiscal processes are in place; that was the most challenging part of the process. (Interview 8, public official)

I was really feeling hopeful about what [the housing initiative] could become, and I still have some hope. But it's becoming jaundiced by how they set up the reimbursement, what you have to do in order to reimburse for services and what that meant … to be able to pull that off without losing money. (Interview 17, homeless services provider)

To implement the tenancy support demonstrations, public agencies had to contract with homeless services providers and get them certified in order to receive payment from Medicaid. In turn, these providers had to perform services that Medicaid officials considered reimbursable; they had to follow standard operating procedures to document their activities though invoices and other means in order to get paid. Some agencies sought to minimize this challenge by contracting with larger nonprofits with experience in billing Medicaid. The demonstrations frequently, however, would partner with smaller community‐based nonprofits without Medicaid experience.

The stakeholders cited several challenges associated with contracting and payment.^33^ One cluster of problems pertained to the formal contracting processes. Government contracting procedures designed to ensure competitive bidding and prevent abuse can lead to delays. As one local official explained,

We are trying to write an RFP to go out to bid for a supportive housing provider… . We have an RFP process that can take up to, easily, six months… . So, I am trying to find any avenue outside our county procurement system… . That has become quite a task for us to figure out. (Interview 23)

Intergovernmental dynamics sometimes lead to contracting complexities. In California, certain counties contracted with city governments within their jurisdictions, which then subcontracted with nonprofits to provide tenancy services. Noting the delays in getting this network up and running, one provider warned, “Frankly, I would never recommend … something like this because the state's now paying the county to pay the city to then pay … nonprofits, and so we have had to deal with bureaucratic requirements at each of those levels” (Interview 20). A local official echoed this sentiment: “It takes us a really long time to get any contract money out and requires a lot of pushing. Although people identify how homelessness is an emergency, it doesn't mean that they're willing to suspend any of their [contracting] processes to be able to move faster” (Interview 11).

The procedural requirements for housing nonprofits to become certified for Medicaid payment also proved taxing and, at times, led to delays. One homeless services provider noted that “going through the basic Medicaid application to get a provider number is a big deal” (Interview 10). Another underscored the time it took to get certified by Medicaid:

There's a lot of phone calls. Signing up on a new online system. I had to have my executive director talk to somebody on the phone. We had to get a special IRS form that our chief financial officer had to help me get. So there were a bunch of steps along the way that were just not simple. (Interview 21)

Once they were certified by Medicaid, billing and getting paid also proved challenging for many housing services nonprofits. One state administrator summed it up as follows: “Another lesson that we've learned is that a lot of these [housing nonprofits] don't understand Medicaid as insurance and that there [are] a lot of documentation requirements.” They “historically just received grant funding or local funding and not had the bureaucratic requirements that come with … insurance” (Interview 22). To be paid for their claims, the providers must document the authorized services they have delivered. For example, under a commonly used “per‐member‐per‐month” payment system, homeless services providers generally had to describe the number of contacts with the client, the services delivered, and when. For instance, one demonstration required the delivery of three eligible services to the client during the month to justify payment. At times, tenancy support providers had to go to considerable lengths not only to demonstrate that their services were medically necessary but also to show that they were not already covered by another funding source. Many of the participating providers were running several housing initiatives and had to establish that Medicaid payments were not substituting for tenancy support services subsidized by other funders. In Washington State, several respondents referred to this as the “supplantation” challenge.

Problems in locating clients sometimes make it difficult for homeless services providers to meet Medicaid's service requirements. The technical skills of frontline outreach workers may also impede efforts to document services. Many demonstrations rely on workers with “lived experience” to track and serve their clients. In their former lives, these employees did not have housing, had behavioral health issues, had been imprisoned, or had other experiences that give them firsthand insights into the challenges people experiencing homelessness face. But as one provider observed, these community workers “may not have graduated from high school” and “may not have all the technical skills that would be required to put in case notes and to navigate” the reporting system without extensive training (Interview 5). Still other payment delays result from invoices that stray from Medicaid's technical requirements for submitting such information. State Medicaid agencies have tried to ameliorate payment problems for homeless services providers through orientation and training. But this remains a work in progress.

These and related factors mean that many Medicaid payment claims submitted by homeless services providers are denied, at least initially. The comments of one such provider conveys the frustration this can create: “I can't stress how ridiculous it's been, so a one‐month invoice may have 14 different edits going back and forth until it's been approved” (Interview 20). Experience with claims denials has prompted some homeless services providers to become more conservative in providing initial services to the homeless. Rather than intervene immediately on the assumption that Medicaid will pay them, they seek preauthorization. As one provider put it, “We had clients that we had provided several hours’ worth of services to … later … find out [our claims] had been denied. We had to go back and say … wait, we need to not continue serving until we get that first approval” (Interview 10). Such delays risk having the provider lose track of the client's location.

In addition to expressing concerns about the administrative burdens of getting paid, housing services providers frequently questioned the adequacy of Medicaid's payments. One provider complained that government contracts tend to “shave us as close to the bone as possible” (Interview 21). Other concerns were with start‐up funding and overhead rates. With respect to the former, one homeless services provider noted that government contracts often forced nonprofits to “carry one to three months of cost waiting reimbursement. It's simply not sustainable” given the limited rate of return on these contracts (Interview 25). As for overhead, the same provider noted that it was “a big uphill battle with government to get them to recognize full program costs, which include … direct funding for costs that are sometimes shoved into the administrative overhead category.” The provider cited data systems essential to the housing initiatives as an example of a cost that government ought to explicitly recognize and fund.

### Challenge 5: Recruiting and Retaining Key Workers

I mean all of us who have been involved, including the workers, have joked about the feeding frenzy of trying to hire community health workers … everyone and their uncle is trying to hire community health workers, people with lived experience who can help really meet the care needs of these vulnerable populations. (Interview 4, consultant)

In addition to involving the usual spectrum of health care professions, the tenancy support initiatives tend to rely heavily on case managers and community health workers. Case managers seek to coordinate the medical, behavioral health, housing, and related social services that enrollees receive. Employees with social work degrees frequently perform this role. In turn, community workers typically serve on the front lines of these housing initiatives, keeping track of, counseling, and providing tenancy supports to clients. Qualifications for these staff often stress “lived experience.”

Some stakeholders indicated that recruiting and retaining case managers and community health workers posed significant challenges. One county official noted that the demand for the homeless services

workforce has … gone up tremendously and one of the things that's slowing us down right now is … the amount of time it takes our nonprofit community partners that we fund to do this work … to recruit and retain staff… . We are able to get funding to them quicker than they are able to bring their team on board to actually do the work. (Interview 14)

The official pointed to the recruitment of case managers as a particular bottleneck, noting that each time the nonprofit hires a case manager, the county can promptly assign that person 20 new clients.

Another stakeholder attributed the case manager challenge to more basic difficulties of attracting social workers to housing services.

I think social workers in general do not go into the homeless services world… . One of the ways that my organization is trying to address that issue … is trying to make the connection with schools of social work to try and get more people who graduate with a master's in social work to go [into the field]. (Interview 1)

Reinforcing this view, another stakeholder emphasized the importance of employing “the right social worker who's interested” in housing and not “more focused on a different type of social work” (Interview 6). Conditions of employment can cause difficulties in recruiting case managers. One county official said that given the waivers’ five‐year life span, they could hire case managers only as term employees without job security and retirement benefits. In some areas, the dearth of affordable housing makes it hard to recruit them.

Recruiting and training community health workers also presented challenges. These workers often play a significant role in frontline interactions with homeless individuals. For instance, one larger nonprofit pointed out that about half their housing coordinators had at one point been homeless or housing insecure. In California, nearly half of all demonstration enrollees had received services provided by peers.^19^ ^(p26)^ A key issue with respect to these coordinators, many of whom lack high school diplomas, centers on training. In this regard, one stakeholder worried that “timeline” was a challenge—whether “we can get these people [with lived experience] trained and into the system quickly enough” to enable the pilot to avoid delays (Interview 1). The demand for workers with lived experience has prompted local educational institutions to offer special training programs. For instance, Los Angeles Southwest College launched an initiative called Careers for a Cause, an eight‐week training program, to prepare individuals with lived experience for community work.^34^ More commonly, the demonstrations have promoted specific training to enable community housing coordinators to perform such functions as taking case notes and entering data into the pertinent information systems. The criminal record of some employees with lived experience at times complicates recruiting and assigning them work (for instance, engaging with clients at risk of becoming homeless upon their release from prison).

### Challenge 6: Durability Beyond the Current Waiver

I think most counties assume there's gonna be a cliff at the end of [the waiver] and that federal funding will go away. (Interview 1, homeless services provider)

Right. We call it the Cinderella problem; everyone is going to turn into a pumpkin [then]. (Interview 2, consultant)

What we promised the state is that we would build a program that is sustainable and that we will work with providers to get them ready to be transitioned. (Interview 13, health care provider)

Medicaid's tenancy support waivers last for five years, and many stakeholders worry about the sustainability of their initiatives beyond that time. Some respondents, however, found certain paths forward. First, CMS might renew the waivers, perhaps after negotiating modifications, which has been an increasingly common practice since the Clinton administration. Second, a few officials believed that local funds might sustain the housing support initiatives. For instance, several California localities passed tax measures to fund homeless services, which might be tapped if the waivers ended. Third, some stakeholders believed that Medicaid's tenancy supports for the homeless population could be preserved through a state plan amendment under Section 1915(i) of Medicaid law. (We will discuss this provision in more detail later.)

Finally, and of particular importance, several stakeholders saw the future of housing supports as dependent on whether Medicaid MCOs would provide these services under their state contracts. The degree to which MCOs would do so was an open question among our respondents. In general, stakeholders in California praised the engagement of MCOs in supporting the demonstration. Some MCOs in that state have invested their own resources in housing supports independent of Whole Person Care. In Maryland, the nine Medicaid MCOs have not actively participated in the demonstration partly because of concern about the “wrong pocket problem”—fear that someone else will get the savings that an MCO creates. One tenancy support provider observed that “it usually goes something like this” when approaching an MCO:

“Here's how many hospital visits … [homeless people] have had. Help us move these people into housing and let's watch what happens with your health care costs.” And you have some MCOs that really believe that… . And then they say, “But how can you guarantee those savings are going to accrue to us? What if the person changes MCOs and goes to another one and then all that work we have done, the benefit will go to somebody else.” (Interview 9)

In Illinois, some Medicaid MCOs had met with homeless services providers and supported the demonstration waiver, but they did not vigorously lobby state Medicaid officials to move forward with the pilot.

Among the four study states, Washington most clearly envisioned that MCOs would lead in ensuring the sustainability of tenancy supports. The contract with Amerigroup as the third‐party administrator (one of five Medicaid MCOs) rested on the premise that it would secure the other MCOs’ short‐ and long‐term commitments to housing supports. As one housing services provider told us, “The goal was for the [housing] benefit to be rolled into all five MCO contracts … at the end of the demonstration” (Interview 19). Such a step could help ameliorate the wrong pocket problem rooted in MCO competition for Medicaid enrollees. No Medicaid MCO could readily gain a competitive advantage over another by failing to offer the housing benefit.

### Challenge 7: Administrative Crowd‐Out and Waiver Burden

So I think when the new administration took office in January, there were other priorities… . There was a tremendous backlog of Medicaid applications and redeterminations… . That became the top priority for [state Medicaid officials] and rightfully so. (Interview 26, homeless services provider)

While Medicaid officials in California, Maryland, and Washington faced significant challenges, by mid‐2020, all three programs had made substantial headway in implementing the housing demonstrations. In contrast, Illinois had not. The administration of Republican Governor Bruce Rauner, which had submitted the waiver proposal, had won CMS approval for it in July 2018. As an initial step, it encouraged the formation of the 12‐member Chicago and Cook County Housing for Health Work Group, consisting of housing and behavioral health providers as well as MCOs and advocates. This group submitted its implementation recommendations to state Medicaid officials in December 2018. By that time, however, Governor Rauner had lost his bid for reelection and Democratic Governor J.B. Pritzker was about to take office in January. The gubernatorial transition featured a substantial turnover of top Medicaid officials, with several of those who had helped secure the waiver departing. The stakeholders attributed the substantial delay in implementing the demonstration to two primary factors: administrative crowd‐out and the implementation burdens associated with waivers as a policy tool.

Administrative crowd‐out refers to the degree to which a given item, in this case Medicaid tenancy supports, gets displaced from an action agenda by issues that officials deem more pressing. The stakeholders identified one major crowd‐out problem affecting Illinois's waiver initiative. As the immediately preceding quotation indicates, the Pritzker administration inherited severe troubles with the state's Medicaid eligibility determination system. Other stakeholders reinforced this view. One health care provider explained that in Illinois,

we have a new eligibility system that has not worked for the last two and a half years … and caused massive problems … with Medicaid initial application delays and also redetermination. It's led to a lot of churn. It's led to providers not being able to get paid. (Interview 27)

Yet another stakeholder perceived the eligibility system's “very onerous Medicaid redetermination process” as a key source of the problem: “It's definitely much easier to get cut off of Medicaid than to be enrolled” (Interview 28).

Second, the stakeholders pinpointed the implementation burdens of waivers as a major factor undermining takeoff. The Rauner administration had been attracted to a Section 1115 waiver in part because it allowed the state to confine tenancy supports to certain geographic areas and to cap enrollments. In contrast, offering these benefits through a state plan amendment under Section 1915(i) of the Medicaid law would designate them as a statewide, open‐ended entitlement for individuals who met certain eligibility criteria. According to the stakeholders, however, the Pritzker administration saw CMS's waiver requirements as “extremely arduous” (Interview 26). The newly appointed Medicaid officials agreed with this view: “We don't really need to demonstrate ACIS in an 1115. We already know that it is a service that can be done… . We can use other Medicaid fiscal levers, like 1915(i) to deliver the same service” and avoid the “onerous … reporting requirements and the budget neutrality” mandate that comes with the demonstration waiver (Interview 28). They could also avoid the resource commitment required to formally evaluate the waiver. These factors led state Medicaid officials to approach CMS about the feasibility of pursuing a 1915(i) state plan amendment to replace the waiver.

## Early Returns

Despite the seven challenges just documented, preliminary evidence suggests that California, Maryland, and Washington have made considerable headway in implementing their waivers. To be sure, we lack comparative data from the three states on such pertinent indicators as the number of people receiving different levels and kinds of tenancy supports as well as their housing status. But some evidence is available. The 25 California pilots enrolled a cumulative total of 108,667 unique individuals in Whole Person Care in 2017/2018. About 43% of these enrollees were experiencing homelessness. As of 2018, about two‐thirds of this cohort had received tenancy supports.^19^ ^(pp25,32)^ Compared to California, Maryland from the outset had targeted fewer individuals for housing supports, about 300. A waiver amendment that CMS approved in April 2019 allowed the state to double that number to 600. As of December 2019, 242 individuals were receiving tenancy supports through the participating localities.^35^ ^(p8)^ In Washington State, the Section 1115 waiver proposal had anticipated a monthly enrollment of 7,500 people in its Foundational Community Supports initiative to provide employment and/or housing supports to Medicaid enrollees. As of September 2019, the initiative had come close to that goal with 6,925 current enrollees, 3,625 of whom were receiving tenancy supports.^36^

## Lessons

The implementation designs and challenges we discussed here point to lessons for other states interested in Medicaid tenancy supports as a pathway to more cost‐effective health care for people experiencing or at risk of homelessness. Three especially stand out.

*First, the two major implementation designs we examined, bottom‐up intergovernmental and third‐party administrator, both show promise as vehicles for promoting Medicaid housing supports*. Early returns from California and Maryland suggest the merits of the locally driven model. Under this approach, state Medicaid officials issue an RFP directed at local governments and count on them to come up with innovative, effective ways to proffer tenancy supports. In doing so, the states would relinquish some control in that localities with significant homeless problems may decline to participate. This did not occur in either California or Maryland, where populous localities with significant homeless problems submitted proposals that the state approved. Reliance on this bottom‐up model has several advantages for state officials. For one thing, they can be reasonably confident that local implementing agents have a strong commitment to the initiative. Localities have some “skin in the game” in that they contribute the state's share of Medicaid funding to draw down federal dollars. In order to be compensated fully for their initial investment, localities have a strong incentive to get the tenancy support program up and running. Compared to their state counterparts, local officials typically have closer relationships with community stakeholders and more expertise regarding the complex forces driving homelessness and affordable housing issues in their jurisdictions. The locally driven model also affords significant opportunities for policy learning. Both California and Maryland have encouraged the formation of learning collaboratives in which the state convenes meetings of local implementers to facilitate communication about what works and what does not.

The bottom‐up intergovernmental model has some drawbacks as well. In California, for instance, it has sometimes heightened the transaction costs of implementation as counties contracted with cities within their boundaries, which in turn subcontracted with providers of tenancy supports. The model is also dependent on waivers because it does not meet the Medicaid legal requirement that benefits be offered statewide. The ability of state officials to continue the initiative depends on CMS's periodic review and approval. Waiver review processes tend to be more exacting than those applied to Medicaid's state plan amendments.

Washington's third‐party administrator approach also has advantages. It allowed the state's Medicaid agency to reduce greatly the administrative burdens of the waiver. Officials did not have to recruit local governments or myriad homeless services providers to implement the program. The model is also less waiver dependent than the intergovernmental approach in that it offers benefits statewide. This could make it easier for officials to sustain tenancy supports once the waiver ends by making it part of their Medicaid MCO contracts. The stakeholders indicated that the choice of Amerigroup as the third‐party administrator created some confusion among providers because they were used to dealing with the entity as one among five Medicaid MCOs. But this selection also meant that Amerigroup would build its capacity to implement a tenancy support initiative if Washington state officials made it part of their MCO contracts.

*Second, states interested in developing Medicaid's tenancy support initiatives need to anticipate and bridge the distinct differences in perspectives and experiences embedded in the health care and homeless services silos*. Large numbers of those we interviewed underscored the challenges of the silos. A major issue was acquainting the providers of tenancy supports in homeless services organizations with the Medicaid reimbursement model. Ostensibly straightforward tasks of getting homeless services providers certified by Medicaid and promptly paid often proved difficult.^37^ Community workers with lived experience often lacked the background needed to meet Medicaid's requirements for documenting services. Rejected claims increased the fiscal stress for homeless services providers, many of whom found the Medicaid payment rates to be minimal in the first place. In turn, health care providers sometimes failed to grasp how the diverse health problems of those experiencing homelessness complicated their efforts to find them housing that would accommodate their particular needs. These and related experiences suggest the importance of procedural and structural steps for overcoming the silos challenge. While sustaining procedural changes in recruitment and training may be challenging in resource‐constrained organizations, the stakeholders emphasized the importance to homeless services providers of intensive training for Medicaid payment requirements. They also embraced structural changes, including the establishment of steering committees and more general governing structures that mandated participation by homeless services, health care, criminal justice, and other stakeholders, as well as frequent interaction among them. Such changes might range from broad cross‐sector integration strategies like the Accountable Communities for Health model^38^ or less formal interorganization workgroups. In addition, the stakeholders brought up the potential value of information systems that integrated health care and homeless services data to break down silos. Achieving effective data integration can be difficult, given the complex privacy regulations, questions of funding, and technical considerations.

*Third, states interested in Medicaid's housing support initiatives should weigh the advantages and disadvantages of Section 1115 waivers relative to other policy tools like state plan amendments*. Section 1115 demonstration waivers comprise one tool for advancing Medicaid's housing supports. But as the case of Illinois suggests, pursuing tenancy supports through a state plan amendment also deserves consideration. The chief advantage of Medicaid's demonstration waivers is the flexibility they offer states to transcend the Medicaid statute in determining who gets what, when, and how from the program. For instance, they can allow states to cap enrollments rather than entitle all individuals who meet certain eligibility criteria to receive benefits; they can target services to certain geographic areas rather than offer them statewide. Section 1115 waivers also provide an opportunity for evidence‐based policy learning in that they require formal independent evaluations of the demonstrations.

But these waivers also have potential downsides for state officials. Those developing and seeking approval of demonstration waivers face substantial transaction costs. Among other things, waiver proposals must undergo significant review and comment periods at both the state and federal levels. While this practice serves the interests of transparency, and stakeholders’ comments may galvanize improvements in the proposed waivers, these process requirements consume significant amounts of time and administrative resources. Negotiations with CMS over waiver specifics are frequently protracted as well. In addition, the five‐year time limit on waivers may make it difficult to hire the staff needed to implement them.

The requirement that demonstration waivers be budget neutral can pose other challenges. The rules for estimating budget neutrality are far from set in concrete. When a presidential administration sympathizes with the goals of a waiver, CMS has often accepted optimistic state assessments of a waiver's budget neutrality.^31^ In the case of the housing support demonstrations, however, concerns about budget neutrality may become salient in CMS's decisions on whether to continue the waivers. States will face pressure to determine whether the extra monies spent on tenancy supports will be offset by lower health care expenditures for people experiencing homelessness. Demonstrations that fail to meet that standard may face CMS's resistance to renewing them even if they can show that those targeted for tenancy supports experience better health outcomes.

In addition, state officials may see the price tag of the formal evaluations as a downside to demonstration waivers. In March 2019, CMS issued guidance likely to increase evaluation costs. The new guidelines call for more complex evaluation designs, new data requirements, and earlier evaluation planning. States bear 50% of the costs of these evaluations, with the federal government covering the rest.^39^

Waiver considerations like these have prompted some state officials to consider providing tenancy supports through a state plan amendment. Section 1915(i) of Medicaid law looms large in this regard. Created by the Deficit Reduction Act of 2005 and amended by the Affordable Care Act in 2010, this provision allows state Medicaid programs to offer home‐ and community‐based services to nonelderly adults with mental health problems, SUD, or other chronic conditions, without having to show that it reduces the program's institutional care costs (e.g., for nursing homes). This target population may include those experiencing or at risk of homelessness. Those states under Section 1915(i) do not have to show that their housing support interventions are budget neutral or subject them to formal evaluation.

Section 1915(i), however, does constrain states in two major ways that demonstration waivers do not. First, it does not allow states to contain costs by capping enrollment. As a result, this compels those states interested in controlling enrollment to do so by adjusting the clinical eligibility criteria for participation in the program, which is often a more uncertain and administratively cumbersome approach and one that may raise concerns about fairness. Second, Section 1915(i) requires that the tenancy support benefit be offered statewide. This places barriers in the way of those states seeking to fine‐tune their tenancy support benefits by phasing them into certain areas before introducing them across the entire state.^40^

## Policy Implications

Maybe [policymakers] have some … perspective that just makes them fearful that Medicaid becomes this bottomless pit of solutions to the world. I can understand that point of view, but the fact is, if we can't loosen up the way certain things are paid for, we're not gonna move people efficiently from the streets to housing. (Interview 17, homeless services provider)

Doctors should be able to write prescriptions for housing the same way that they do for insulin and antibiotics. (Governor Gavin Newsom of California in his State of the State address, 2020)^41^

This assessment of four state demonstrations also raises the question of whether Medicaid policy should be altered to better address housing as a social determinant of health. The call to bolster Medicaid's role in addressing housing needs partly reflects the shortcomings of current federal, state, and local efforts to resolve problems of affordable housing and homelessness. In the 1970s, the housing market had a small surplus of rental units to house the poor. More recently, though, this surplus has been transformed into an estimated shortage of 7.1 million rental units for people with very low incomes.^42^ Incentives for builders to construct “deeply affordable” housing have generally failed to stimulate such activity. Nor have myriad federal programs been able to fulfill the demand for housing among people at risk of or experiencing homelessness. While the Veterans Administration has made significant progress in providing housing supports for its clientele, the supply of subsidized public housing has, in general, shrunk.^43^ HUD's HOPE VI program, created in 1993, made grants available to local public housing authorities to demolish severely stressed housing projects and replace them with redesigned mixed‐income neighborhoods. Originally, federal policy required that any public housing unit lost be replaced through a voucher or other means. But in 1995 Congress repealed the one‐for‐one replacement rule, allowing public housing authorities to demolish more units than they created. More generally, Section 8 vouchers and other federal housing assistance to low‐income households serve an estimated quarter of the eligible renter population.^44^ More recently, HUD has, under the banner of promoting racial justice, sought to incentivize continuum‐of‐care entities to assess their systems for racial discrimination and to remedy any deficiencies.^45^ But it remains to be seen whether this will help the disproportionate numbers of African American people experiencing homelessness.

At the local level, inflexible, antigrowth, and exclusionary zoning codes often inhibit housing initiatives. In some cases, elaborate and costly housing permit requirements and environmental regulations pose additional obstacles.^46^ Widespread individual preferences for homogeneous neighborhoods, along with concerns about property values, school capacity, and public safety, frequently strengthen the NIMBY forces.^47^, ^48^, ^49^The attraction to local elected officials of economic development initiatives rather than redistributive actions that benefit lower‐income people also inhibits support for affordable housing.^50^ To be sure, local policymakers have sometimes allocated public funds to address homelessness in their jurisdictions. In California, for example, voters in several counties have approved tax increases for this purpose. While helpful, the sums of money involved permit only marginal progress in fighting homelessness.^51^ ^(p156)^

State policymakers have at times tried to override local policies and practices that inhibit efforts to develop affordable housing and reduce homelessness. Over the years, for instance, certain California policymakers have repeatedly sought to overrule local density, building height, and other restrictions to foster the construction of apartment buildings near train and bus transit stops.^51^ In fiscal years 2019 and 2020, the state committed $1.5 billion to help local governments cope with homelessness.^41^ To a large degree, however, the leverage to address the problems of affordable housing and homelessness continues to reside at the local rather than the state government level.

Compared to housing, policy dynamics at the federal and state levels have been much more conducive to program durability and growth in the case of health care in general and Medicaid in particular.^31^, ^52^ The supportive political constituencies that Medicaid has attracted over the decades have been much more formidable than those arrayed behind initiatives to supply housing for low‐income people. Medicaid's relative political strength has heightened its appeal to address the housing needs of those experiencing homelessness with more acute health problems.

Various stakeholders have called for policy changes that would allow Medicaid to subsidize rents for those experiencing or at risk of homelessness. Among other things, they argue that the “medicalization” of housing offers an integrated approach to addressing health problems that clinical interventions alone cannot solve and will motivate health care providers to identify new partners to advance the public's health.^53^ Specific proposals vary. One observer would exclude from the benefit those experiencing short‐term homelessness and instead would target those with mental health problems or SUD who are physically capable of performing the activities of daily living but unable to secure stable housing.^54^ Another recommends that state Medicaid officials rely on a vulnerability index to target for rental subsidies those experiencing homelessness with the most acute health problems. This index would incorporate these individuals’ diagnostic codes and past utilization patterns. States lacking the capacity to develop such an index could implement an alternative “strict approval process and an ongoing review mechanism” to determine eligibility for Medicaid rental subsidies. Under this plan, Medicaid MCOs would have the authority to pay for supportive housing.^55^

A definitive assessment of the potential cost‐effectiveness of expanding Medicaid's authority to subsidize rental costs for those experiencing homelessness falls beyond our ken. Still, certain implications of any such change deserve note. If federal policymakers authorized direct Medicaid rental subsidies in response to homelessness, it would likely be an optional rather than a required benefit. The degree to which states would choose to offer it remains a very open question. The states that have not expanded Medicaid under the Affordable Care Act would not be in a position to do so. For expansion states, cost considerations would likely loom large. While interested states could count on the federal Medicaid match to subsidize their initiatives, and some states would receive a 90% match, many would still need to come up with an appreciable amount of funding. This would add to the strain that Medicaid already places on many state budgets. In addition, the economic recession induced by the COVID‐19 pandemic would further compound the fiscal stress associated with such a step, at least over the short term. Cost concerns would grow if the rental subsidy option required that the benefit be offered statewide without enrollment caps. Of course, these concerns would fade if the costs of housing subsidies were offset by reduced emergency department, hospital, and other medical utilization among those experiencing chronic homelessness. Given the uncertainties about net costs and other factors, states might still prefer to test the implications of direct rental subsidies through Section 1115 waivers rather than immediately offer the option through a state plan amendment.

## Limitations

Care should be exercised in generalizing our findings to other states. We studied four early‐adopter states, each operating in unique circumstances. California and Washington, for example, have long struggled with highly visible and persistent street homelessness, which no doubt gave impetus to their initiatives. The policy context, service infrastructure, and demographic circumstances of states vary widely, and while some of the states we did not study may draw specific lessons from our findings, broad generalizations are not possible. Furthermore, while our key informant recruitment strategy was systematic and thorough, and our interviews were in‐depth, there may be other questions we could have asked or additional stakeholders to interview whose perspectives could have enriched our findings. Nevertheless, our interviewees had rich and diverse perspectives, and we employed strategies to minimize researcher bias (e.g., consensus coding), thereby enhancing the transferability of our findings to other contexts.

## Conclusions

During the past several years, various stakeholders have encouraged health care providers to address the social determinants of poor health and the potentially avoidable use of expensive medical services. Among these determinants, adequate housing figures prominently. Findings about the relationship between housing and health care utilization have encouraged Medicaid officials in a growing number of states to consider providing tenancy supports to those experiencing or at risk of homelessness. This article examined the implementation challenges faced by four early‐adopter states (California, Illinois, Maryland, and Washington) that sought to provide tenancy supports through Section 1115 Medicaid waivers. Despite facing significant implementation difficulties, including limitations on the availability of affordable housing, problems with coordinating the work of state Medicaid programs with housing providers, complications of enrolling people in the programs, the difficulty of recruiting pertinent staff, and worries about program durability, three of the states made significant progress in launching their initiatives. A fourth state, Illinois, has delayed its start‐up in order to consider alternatives to a Medicaid demonstration waiver as a vehicle for tenancy supports. The experience of the four waiver states suggests lessons for Medicaid officials in other jurisdictions who are interested in pursuing housing support initiatives. Nevertheless, the limitations of the tenancy support waiver programs mean that federal policymakers should consider allowing states to directly subsidize housing for those experiencing or at risk of homelessness as an optional Medicaid benefit.

*Funding/Support*: Support for this research was provided by the Robert Wood Johnson Foundation. The views expressed here do not necessarily reflect the views of the Robert Wood Johnson Foundation.

*Acknowledgments*: We gratefully acknowledge the invaluable input of our stakeholder interviewees, our state consultants, staff at AcademyHealth, and our Milbank reviewers. We also thank our Rutgers colleague Bram Poquette for assistance in preparing this manuscript.

*Conflict of Interest Disclosures*: All authors completed the ICMJE Form for Disclosure of Potential Conflicts of Interest. No conflicts were reported.
